# Implication of Enterohepatic Re-Circulation on Single Dose Bioequivalence Evaluation of Two Brands of Clonidine Hydrochloride Tablets in Healthy Human Volunteers

**DOI:** 10.4103/0250-474X.58181

**Published:** 2009

**Authors:** H. R. Mehta, I. K. Patel, N. H. Patel, D. M. Patel, A. B. Parmar

**Affiliations:** Clinical Research Group, Torrent Research Centre, Gandhinagar-382 424, India; 1 Clinical Pharmacology and Pharmacokinetic Unit, Cadila Pharmaceuticals Ltd., Dholka-387 810, India; 2 Department of Pharmacology, S. K. Patel College of Pharmaceutical Education and Research, Mehsana-382 711, India

**Keywords:** Enterohepatic re-circulation, clonidine hydrochloride, area under curve, analysis of variance, bioequivalence

## Abstract

A single dose, crossover bioequivalence study of two different brands of clonidine hydrochloride 25 μg tablets was conducted in 24 (+2 stand by) healthy, adult, male, Indian subjects under fasting conditions to check the implication of enterohepatic re-circulation on assessment of bioequivalence. After an overnight fasting of at least 10 h, the subjects received single oral dose of test or reference product with either of the product as per randomization schedule in each period with a washout period of 10 days. The pre-dose blood sample was collected within a period of one h before dosing. The post-dose blood samples were collected at specified time intervals up to 96 h. The plasma concentrations of clonidine were quantified by validated LCMS/MS method and pharmacokinetic parameters were computed. The 90% confidence intervals of test/reference ratios for C_max_ and area under the plasma-concentration- time-curve AUC under 0-t were found to be between 0.80 and 1.25 for log-transformed data. Analysis of variance did not show significant difference to these parameters. No meaningful values of K_el_ and therefore AUC under 0-infinity could be calculated for significant number of subjects due to enterohepatic re-circulation. Based on the results obtained, two different brands of clonidine 25 μg tablets have comparable rate and extent of absorption after oral administration but failed to show bioequivalence as per regulatory requirement of Food and Drugs Administration-united states.

Clonidine is an antihypertensive agent, which acts centrally by stimulating alpha_2_-adrenergic receptors and producing a reduction in sympathetic tone, resulting in a fall in diastolic and systolic blood pressure and a reduction in heart rate. Treatment with clonidine diminishes the responsiveness of peripheral vessels to constrictor and dilator stimuli, thereby preventing the vascular changes associated with migraine. Clonidine, as an imidazoline, binds to imidazoline receptors (I_1_, I_2_, I_3_), in addition to its well described binding to α_2_ receptors. Two newer antihypertensive imidazolines, rilmenidine and moxonidine, have profile of action similar to clonidine, suggesting a role for I_1_ receptors. However, the lack of an antihypertensive effect of clonidine in knockout mice lacking α_2A_ receptors supports a key role for these receptors in blood pressure regulation[1,2]. Clonidine stimulates the α_2A_ subtype of α_2_-adrenergic receptors in the brainstem, resulting in a reduction in sympathetic outflow from the CNS[1]. The decrease in plasma concentrations of norepinephrine is correlated directly with the hypotensive effect[3]. At doses higher than those required to stimulate central α_2A_ receptors, this drug can activate α_2_ receptors of the α_2A_ subtype on vascular smooth muscle cells[1,4]. This effect accounts for the initial vasoconstriction that is seen when overdose of clonidine taken and it has been postulated to be responsible for the loss of therapeutic effect that was observed with high doses[5].

The plasma level of clonidine peaks in approximately 3 to 5 h and the plasma half-life ranges from 12 to 16 h. The half-life increases up to 41 h in patients with severe impairment of renal function. Following oral administration about 40-60% of the absorbed dose is recovered in the urine as unchanged drug in 24 h. About 50% of the absorbed dose is metabolized in the liver[6]. The impact that enterohepatic cycling (EHC) has on the pharmacokinetics and pharmacodynamics of a drug depends on the importance of biliary excretion of the compound relative to renal and metabolic clearance processes and on the efficiency with which the compound is absorbed into the circulation from the gastrointestinal tract. when the compound is well absorbed and biliary secretion is a major clearance mechanism, EHC becomes prominent. As a result, a major fraction of the absorbed dose in the n^th^ cycle participates in the (n+1)^th^ cycle, leading to a cumulative body exposure to drug which may exceed the dose by several fold. In this context, EHC has a unique impact on both the pharmacokinetics and the pharmacodynamics of the compound[7].

Various pharmacokinetic studies confirm enterohepatic re-circulation of clonidine after oral administration. Special attention should be drawn to the unexpected findings like, late increase in the declining plasma levels and the non-linear relationship to dose of the absolute plasma concentration of clonidine. Both phenomenons are probably signs of the enterohepatic circulation of clonidine, which is already demonstrated earlier. As the pharmacokinetics of clonidine are most probably influenced by enterohepatic circulation, the kinetics cannot be described by conventional open one- to open two-compartment model, and these more complex process require a specially adapted multi-compartment model[8]. Enterohepatic re-circulation; being known characteristic of clonidine, the present study was designed to demonstrate the implication of it on assessment of bioequivalence of two different brands of clonidine.

## MATERIALS AND METHODS

The present study was conducted in 26 healthy adult volunteers (males), with a target age between 18-45 years having a body mass index (BMI) between 18-25 kg/m^2^, who had no evidence of underlying disease or significant abnormal laboratory at screening and who voluntarily consented to participate in the study. The study was performed in accordance with the latest revisions to the Declaration of Helsinki, ethical approval having been obtained from the Independent Ethics Committee (WMA General Assembly, Tokyo, Japan 2004) and Health/Regulatory Authority. All volunteers gave their written informed consent prior to commencement of the study. There is no financial and personal relationships exist between the authors and others that might bias their work. No potential conflicts exist between authors. Independent Ethics Committee approved the study protocol prior to initiation.

### Study design:

This was a randomized, open label, two treatment, two period, two sequence, single dose, two-way crossover, bioequivalence study of test product of clonidine hydrochloride 25 μg tablets and reference product of clonidine hydrochloride 25 μg tablets, in healthy human adult male subjects, under fasting conditions. The subjects were administered a single oral dose of either the test or reference product of 25 μg clonidine tablet. To eliminate the carryover effect of the drug, there was a washout period of 10 days between the period I and the period II. In each period, a total of 19 blood samples (5 ml each) were collected, prior to drug administration at 0.00 and after drug administration at 1.0, 2.0, 2.5, 3.0, 3.5, 4.0, 4.5, 5.0, 6.0, 8.0, 10.0, 12.0, 16.0, 24.0, 36.0, 48.0, 72.0 and 96.0 h. The total volume collected per subject in this study was not exceeded 220 ml including 8-10 ml for screening, 5.0 ml for post study safety analysis and 0.5 ml of ‘discarded’ anticoagulant mixed blood prior to each in-house sampling, total volume of discarded blood was not more than 15 ml in both periods. Crossover design was selected in order to minimize the variability by using subjects as their own control. The following precautions were incorporated into the study to minimize bias; 1. subjects were sequentially assigned to randomly ordered treatment, 2. subject enrolment was dependent on satisfactory fulfillment of the given list of inclusion criteria, 3. the circumstances when individual subjects were withdrawn prior to completion of the study were specified and 4. the analyst was blinded to the randomization code of both test and reference product.

### Inclusion criteria:

The inclusion criteria employed were, a. healthy human adult male subjects between 18-45 years of age (inclusive), having a body mass index (BMI) between 18 and 25 kg/m^2^ (inclusive), b. subjects who had no evidence of underlying disease during screening and whose physical examination was performed within 21 days prior to scheduled day for administration of test and reference product, c. subjects whose screening laboratory values were within normal limits or considered by the physician/investigator to be of no clinical significance and d. informed consent given in written form according to protocol.

### Exclusion criteria:

History or presence of significant, a. cardiovascular, pulmonary, hepatic, renal, hematological, gastrointestinal, endocrine, immunologic, dermatologic, musculoskeletal, neurological or psychiatric disease, b. alcohol dependence, alcohol abuse or drug abuse within past one year, c. moderate to heavy smoking (>10 cigarettes/day) or consumption of tobacco product, d. history of difficulty in swallowing tablet or capsule, e. clinically significant illness within 4 weeks before the start of the study, f. asthma, urticaria or other allergic type reactions after taking any medication, g. positive urine drug screening, HIV, VDRL/RPR, Hepatitis B and C tests, h. any history of hypersensitivity to gliclazide or any other sulphonylureas and sulphonamides, i. any history suggestive of hypoglycaemic episode (dizziness, sweating, palpitation, tremor in last 6 months) and j. subject who had participated in any other clinical trial involving drug administration and collection of blood samples or had donated blood in the preceding 12 weeks prior to start of the study.

Subjects were excluded who had: (a) Systolic blood pressure less than 100 mm of Hg or more than 140 mm of Hg. (b) Diastolic blood pressure less than 60 mm of Hg or more than 90 mm of Hg. Minor deviations (2-4 mm Hg) at check-in, if any, were acceptable at the discretion of the physician/investigator. (c) Pulse rate below 60 beats/min or above 100 beats/min

### Dosing:

After an overnight fast of at least 10 h, subjects received single oral dose of test or reference product with 200 ml of drinking water at ambient temperature in each period. Subjects were instructed not to chew or crush the tablets, and were asked to take it with specified quantity of drinking water. Compliance was assessed by conducting a thorough examination of the oral cavity using flashlight and spatula by trained study personnel after dosing in each period and by measurement of plasma clonidine (during the analytical phase of the study). All subjects were dosed at the fixed time and restricted to remain supine for the first 2 h following drug administration. Subjects could be ambulatory and were advised to avoid severe physical exertion for remaining time of the each period. First 2 h post dose samples were collected at bedside and remaining blood samples were taken at sample collection area.

The order of administration of the test and reference products to each subject was determined according to the randomization scheme. The randomization scheme was generated at Statistical unit of CPPU (Clinical Pharmacology and Pharmacokinetics Unit) using SAS Software Version 9.1.3.

### Blood sampling:

Blood samples were withdrawn by an indwelling cannula placed in a forearm vein or (if require) fresh clean venipuncture using a disposable sterilized syringe and a needle in case of clotting of cannula. Blood samples were collected in pre labeled (label mentioning study number, subject number, period and sampling time point) test tubes containing sodium citrate buffer as the anticoagulant and at the times specified under the study design section. After collection and proper mixing, blood samples were centrifuged with the help of refrigerated centrifuge at 4° as soon as possible but not more than 10 min, to separate plasma at 3000 rpm for 10 min. All plasma samples were stored in properly labeled vial, below −20° or colder in deep fridge till analysis.

The pre-dose blood samples were collected within a period of 1 h before dosing and post-dose samples will be within ±2 min of the scheduled time till 24^th^ h blood sample. Ambulatory samples at 36.0 (Day 02), 48.0 (Day 03), 72.0 (Day 04) and 96.0 (Day 05) h post dose were collected with a variation of ±60 min being accepted. The actual mid-point time of collection of each blood sample (to the nearest minute) would be recorded on the appropriate data sheet. At the time of analysis all plasma samples were packed in thermocol box containing coolant bags or dry ice then transferred to the Bioanalytical Laboratory.

### Analysis of plasma samples:

Samples were analyzed for clonidine in plasma using validated LCMS-MS procedures. Whenever possible, all samples from each subject were analyzed on the same standard curve. Standard and quality control samples were included in each batch of study samples assayed. Samples with drug concentrations greater than the upper limit of the validated range of the assay were diluted with the appropriate drug-free biological fluid and re-assayed for those which were below the lower limit of this range were reported as being below LOQ. Repeat assays were done, if required after proper justification and documentation. The analysts would not have access to the randomization scheme.

### Pharmacokinetic analysis:

Pharmacokinetic analysis of the plasma concentrations for clonidine was carried out for the test and reference product to determine the following PK parameters in each individual subject using WinNonlin version 5.0.1. C_max_: Maximum measured plasma concentration following each treatment; AUC_0-t_: The area under the plasma concentration versus time curve from time zero to the last measurable concentration as calculated by linear trapezoidal method; AUC_0-∞_: The area under the plasma concentration versus time curve from time zero to infinity; AUC_% Extrap_: The residual area in percentage (%) determined by the formula, [(AUC_0-∞_-AUC_0-t_)/AUC_0-∞_]×100; T_max_: Time of the maximum measured plasma concentration; T_½_: The elimination or terminal half-life; K_el_: Apparent first order elimination rate constant. No values of K_el_, T_½_ and AUC_0-∞_ were reported for cases that do not exhibit a terminal log-linear phase in the concentration versus time profile.

All concentration values below the limit of quantification (BLQ) were set to zero for all pharmacokinetic and statistical calculations. Any missing samples were reported as ‘M’ and were not included for pharmacokinetic and statistical calculations. Non-compartmental method (Model 200 of WinNonlin) of analysis was used to estimate the above mentioned pharmacokinetic parameters. Linear trapezoidal (linear interpolation) method was used for AUC calculations. The actual time of blood withdrawal was used for pharmacokinetic calculations.

### Statistical analysis:

Statistical analysis was performed using the SAS® software for windows, release 9.1 (SAS Institute Inc., Cary NC, USA) for clonidine. The following summary statistics for the pharmacokinetic parameters were calculated for both the test (A) and reference (B) product: N (No. of Subjects), Arithmetic Mean (Mean), Standard Deviation (SD), Maximum, Median and Percentage Coefficient of Variation (CV%). Additionally Geometric Mean (GM) was calculated for C_max_ and AUC_0-t_. The log-transformed pharmacokinetic parameters AUC_0-t_ and C_max_ were analyzed using generalized linear model procedure of SAS®. The Analysis of variance (ANOVA) model included sequence, formulation (treatment) and period as fixed effects and subjects nested within sequence as a random effect. The sequence effect was tested at the 0.10 level of significance using the mean square of subjects nested within sequence from the ANOVA as the error term, in the F-ratio of the sequence effect. All other main effects were tested at the 0.05 level of significance using the residual error (mean square error) from the ANOVA, as error term in the F-ratio for the respective main effects.

The ratio of the test and reference product averages (Least Square Means) was estimated for the differences in the Least Square Means (LSM) of the log-transformed data then taking the anti-log for the estimates. The 90% Confidence Interval for the ratio of test and reference was estimated using the‘t’ value at Mean Square Error Degrees of Freedom (df), and the Standard Error of Estimate. The Standard Error of Estimate was calculated using the Mean Square Error, and number of reference subjects from the GLM - ANOVA Model.

## RESULTS

**Fig. 1:**
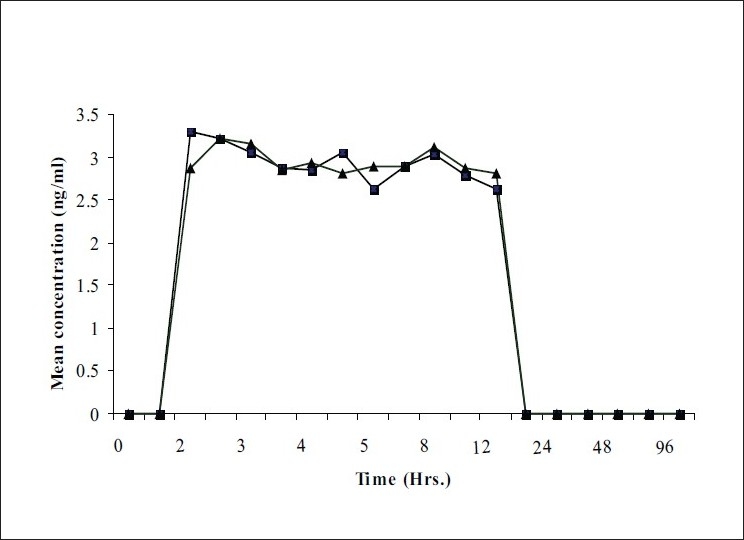
Mean plasma concentration Vs time curve for clonidine (linear scale) Plasma concentrations of clonidine obtained at different time points after oral administration of test product (—▪—) and reference product (—▲—) into healthy human volunteers. Each data point represents mean of n=24.

**Fig. 2:**
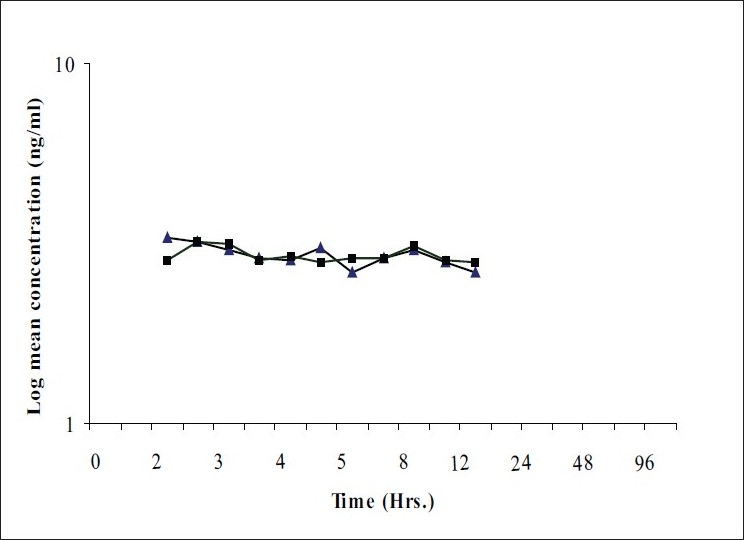
Mean plasma concentration Vs Time curve for clonidine (logarithmic scale) Plasma concentrations (log transformed) of clonidine obtained at different time points after oral administration of test product (—▲—) and reference product (—▪—) into healthy human volunteers. Each data point represents mean of n=24.

The present bioequivalence study was conducted in 26 healthy male volunteers with age between 18 to 30 years and BMI with range 18.07-23.59 kg/m^2^. The final evaluation was carried out on data obtained from 24 volunteers who completed the study according to protocol. The mean plasma concentrations of clonidine for test and reference products on linear and logarithmic scales are shown in figs. 1 and 2, respectively. Detailed demographic data is presented in Table 1.

**TABLE 1:** OVERALL DEMOGRAPHIC PROFILE OF SUBJECTS ENROLLED IN THE STUDY (N=26)

Parameters	Age (y)	Weight (kg)	Height (cm)	Body Mass Index (kg/m^2^)
Mean	23.96	56.22	167.20	20.07
SD	2.93	6.62	6.65	1.65
Min	18.00	48.00	156.50	18.07
Max	30.00	70.00	182.00	23.59

The mean (±SD) C_max_ for the test product (A) was 4.192±1.841 μg/ml and for the reference product (B), it was 3.992±1.363 μg/ml. The mean (±SD) AUC_0-t_ for the test product (A) and reference product (B) were 30.38±10.759 μg.h/ml and 30.89±12.347 μg.h/ml respectively. The median T_max_ was 3 h and 4.75 h for the test product (A) and the reference product (B), respectively. All the main pharmacokinetic parameters are shown in Table 2.

**TABLE 2:** PHARMACOKINETIC PARAMETERS OF CLONIDINE AFTER SINGLE DOSING

Pharmacokinetic parameters	Product	Mean	SD	Minimum	Median	Maximum	CV%
C_max_ (ng/ml)	A	4.192	1.841	2.590	3.810	10.840	43.91
	B	3.992	1.363	2.420	3.750	8.690	34.15
T_max_ (h)	A	3.937	2.420	2.000	3.000	12.000	61.45
	B	5.771	3.220	2.500	4.750	12.000	55.8
K_el_ (h-1)	A	0.039	0.027	0.001	0.038	0.097	70.8
	B	0.069	0.045	0.010	0.060	0.152	66.08
T_1/2_ (h)	A	87.09	259.486	7.154	18.625	1241.62	297.96
	B	18.84	19.129	4.552	11.492	70.356	101.56
AUC_0-t_ ng/ml/h	A	30.38	10.759	17.613	26.884	64.157	35.42
	B	30.89	12.347	18.363	27.885	75.763	39.97

Pharmacokinetic parameters of clonidine after single dosing of (a) test and (b) reference products

### Bioequivalence assessment:

ANOVA for pharmacokinetic parameters as shown in Table 3 (after data logarithmic-transformation) did not show any statistically significant difference between two products. The 90% confidence interval for clonidine log transformed parameters C_max_ and AUC_0-t_ were 99.42 to 111.82 (ratio = 103.30) and 94.76 to 103.52 (ratio = 99.04), respectively, which is shown in Table 4. No meaningful values of K_el_ and therefore T_½_ and AUC_0-∞_ could be calculated for significant number of volunteers due to EHC of clonidine. Values of AUC_0-∞_ were missing for total six volunteers in reference group and two volunteers in test group. Excluding missing data of AUC_0-∞_; the 90% confidence interval (n=16) was found to be 76.89 to 133.05.

**TABLE 3:** TYPE III TEST OF ANALYSIS OF VARIANCE

Statistics	AUC_0-t_	C_max_
Sequence	0.0005	0.1162
Subject (Sequence)	<0.0001	<0.0001
Formulation	0.7117	0.4906
Period Formulation	0.2565	0.4325

Probability-values (Pr>F)

**TABLE 4:** RATIO OF LEAST SQUARES MEAN AND 90% CONFIDENCE INTERVALS OF TEST VERSUS REFERENCE FOR CLONIDINE

Pharmacokinetic Parameter	Ratio of Least squares mean	90% Confidence Intervals
C_max_ (ng/ml)	103.30	99.42 to 111.82
AUC_0-t_ (ng/ml/h)	99.04	94.76 to 103.52

### Tolerability:

Total twenty six volunteers were dosed at least once. All the volunteers were included in the safety evaluation. Of the 26 subjects enrolled, 24 volunteers received both the test and reference products during the study. Total five adverse events were reported in the study out of which; two adverse events (burning sensation and itching in perineum) were reported after test drug administration and three adverse events (fever, bodyache and abdominal pain) were reported after reference drug administration. All the adverse events were mild in nature and resolved completely before discharge of volunteers. There were no trends towards clinically significant changes in laboratory safety parameters.

## DISCUSSION

The main objective of the study was to determine the impact of enterohepatic re-circulation on assessment of bioequivalence of drugs exhibiting the said phenomenon. This study was carried out in accordance with the clinical research guidelines established by the Basic principles defined in the ICH E6 Guidelines for GCP, Indian council of medical research (ICMR) guidelines for biomedical research on human subjects, the principles enunciated in the Declaration of Helsinki (Ethical Principles for Medical Research Involving Human Subjects, revised by the WMA General Assembly, Tokyo, 2004) and Standard Operating Procedures.

The drug concentration levels of clonidine in plasma were determined by a validated LC-MS/MS method. Sampling was done up to 96.0 h after dosing such that the plasma concentrations could be measured for adequately profiling the pharmacokinetics of the product and study periods were separated by a washout period of 10 days for complete elimination of the drug. The pharmacokinetic and statistical analysis was done on 24 subjects out of 26 subjects, excluding subject no. 02 and 13 who were lost to follow up in period II.

Analysis of variance for log transformed pharmacokinetic parameters revealed that there was no significant effect of variation due to period and formulation for all the pharmacokinetic parameters at 0.05 level of significance. The effect of sequence using subjects nested within sequence as error term for all the pharmacokinetic parameters were found to be statistically not significant at 0.10 level of significance.

Results obtained from the study indicated that both test and reference formulation demonstrated comparable rate and extent of absorption. But, for assessment of bioequivalence according to USFDA regulatory requirement, the 90% confidence interval of all three pharmacokinetic parameters i.e. C_max_, AUC_0-t_ and AUC_0-∞_ should fall between 80% to 125%. The present study fails to show bioequivalence if 90% confidence interval of AUC_0-∞_ is taken into consideration. The main reason for non compliance of bioequivalence in terms of AUC_0-∞_ may be enterohepatic re-circulation of clonidine. Further investigations are required to develop more complex modeling for such kind of drugs undergoing EHC during assessment of bioequivalence.

The study results revealed that, the two formulations under the investigation (i.e. Test and Reference) demonstrated comparable rate and extent of absorption in healthy human volunteers under fasting conditions, but failed to show bioequivalence as per USFDA regulatory requirement.
